# In-depth proteome analysis of more than 12,500 proteins in buffalo mammary epithelial cell line identifies protein signatures for active proliferation and lactation

**DOI:** 10.1038/s41598-020-61521-1

**Published:** 2020-03-16

**Authors:** Shalini Jaswal, Vijay Anand, Sudarshan Kumar, Shveta Bathla, Ajay K. Dang, Jai K. Kaushik, Ashok K. Mohanty

**Affiliations:** 1 Proteomics and Structural Biology Lab, Animal Biotechnology Center, National Dairy Research Institute, Karnal, 132001 Haryana India; 2 Department of Veterinary Physiology and Biochemistry, Veterinary College and Research Institute (TANUVAS), Orathanadu, 614625 Tamilnadu India; 3 Dairy Cattle Physiology Division, National Dairy Research Institute, Karnal, 132001 Haryana India

**Keywords:** Protein-protein interaction networks, TOR signalling, Physiology

## Abstract

The mature mammary gland is made up of a network of ducts that terminates in alveoli. The innermost layer of alveoli is surrounded by the differentiated mammary epithelial cells (MECs), which are responsible for milk synthesis and secretion during lactation. However, the MECs are in a state of active proliferation during pregnancy, when they give rise to network like structures in the mammary gland. Buffalo (*Bubalus bubalis*) constitute a major source of milk for human consumption, and the MECs are the major precursor cells which are mainly responsible for their lactation potential. The proteome of MECs defines their functional state and suggests their role in various cellular activities such as proliferation and lactation. To date, the proteome profile of MECs from buffalo origin is not available. In the present study, we have profiled in-depth proteome of *in vitro* cultured buffalo MECs (BuMECs) during active proliferation using high throughput tandem mass spectrometry (MS). MS analysis identified a total of 8330, 5970, 5289, 4818 proteins in four sub-cellular fractions (SCFs) that included cytosolic (SCF-I), membranous and membranous organelle’s (SCF-II), nuclear (SCF-III), and cytoskeletal (SCF-IV). However, 792 proteins were identified in the conditioned media, which represented the secretome. Altogether, combined analysis of all the five fractions (SCFs- I to IV, and secretome) revealed a total of 12,609 non-redundant proteins. The KEGG analysis suggested that these proteins were associated with 325 molecular pathways. Some of the highly enriched molecular pathways observed were metabolic, MAPK, PI3-AKT, insulin, estrogen, and cGMP-PKG signalling pathway. The newly identified proteins in this study are reported to be involved in NOTCH signalling, transport and secretion processes.

## Introduction

The mammary gland, a unique organ of mammals, is a derivative of ventral skin^1^ and has evolved to provide nutrition and immune protection to the offspring. Though the anatomical and physiological features of a mammary gland are diverse, the basic structure is similar in all species. The mature mammary gland is constituted by a network of tubular-alveolar structures, with their lumen lined by a layer of polarized secretory mammary epithelial cells (MECs). The MECs are dynamic components of the mammary gland, which undergo repeated rounds of proliferation and differentiation during different developmental stages such as pregnancy, and lactation^2^. The quantity and functional differentiation of MECs in the udder determine the milk-producing ability in livestock. Bovine (ruminants) are physiologically distinct from other mammals such as humans, mice and rats. The present study on in-depth proteome analysis of buffalo (*Bubalus bubalis*) MECs (BuMECs) advances our knowledge on the protein machinery operating in mammary gland development and lactogenesis in buffalo, which can be extrapolated in other mammalian species as well. Among the domestic livestock species, cattle (*Bos taurus*) and buffalo are the major contributors to the production of nutritious milk for human consumption. India harbours the largest number (57%) of buffaloes in the world that contribute around 51.2% of milk produced in India and 13% of total milk supply in world^3^. Buffalo is the second-largest milk-producing animal, and its milk is rich in fat, solids- not-fat (SNF) and protein content compared to that of cow’s milk^4^. Buffaloes also play an essential role in the development of the economy for the farming community by providing milk, meat, and draft power.

MEC lines from different species serve as a model system to gain insight into the biological functions of the mammary gland. In the present study, we have profiled and analyzed the in-depth proteome of BuMEC line^5^ developed in our lab using a high throughput proteomics approach. Previous studies on global proteome profiling of MECs isolated from milk of cattle^6^ and human (MCF-7)^7^ reported a total of 497 and 3,715 proteins, respectively. Also, various other groups have reported combined proteome from multiple biological sources such as 10,361 ± 120 proteins, each in 11 different human cell lines^8^, 10,350 in NCI-60^9^, and 7,349 in 28 mice tissues^10^. Although the above number of proteins represents a sizeable proteome coverage, recent advancements in the mass spectrometry (MS) have enabled the analysis of complex cellular proteins in high numbers^8^. The main challenge in any proteomic study is to identify the cellular proteins available in minor quantities, which are usually masked by the abundant ones during MS analysis. Identification of proteins from different sub-cellular organelles followed by peptide fractionation and MS analysis is an advanced method for in-depth proteome analysis^11^.

In the present study, we performed in-depth proteome profiling of actively proliferating BuMECs. Ruminants (cattle, buffalo, goat, etc.) constitute a distinct class of livestock species, which are known milk producers. Their physiology is different from other non- ruminant mammalian species such as human, mice, monkey, rat etc. Given the differences in mammary gland physiology between ruminants and non-ruminants, expression of various proteins and molecular pathways associated with proliferation and lactogenesis, mimicking *in vivo* mammary gland development and lactation respectively, may be different in BuMECs. To our knowledge, no information is available on the proteome of BuMECs. Moreover, we have performed proteome analysis of 68 digested protein fractions [24 digested protein fractions for sub-cellular fraction-I (SCF-I); 12 digested protein fractions- each for SCF-II, -III, and -IV; and 8 digested protein fractions for conditioned media] to generate in-depth proteome data, which is the uniqueness of this study. Hence, the present study provides new insights into the molecular physiology of mammary gland associated with its development and lactation.

The primary objective was to identify the maximum number of proteins by adopting advanced methodologies that included sub-cellular protein fractionation followed by peptide fractionation and high-resolution MS. The MS analysis of four SCFs and conditioned media helped us to identify the highest number of proteins reported so far in any mammalian cell line. Furthermore, the Bioinformatics analysis mapped the proteins to various molecular pathways, which may serve as the key regulators for the active proliferation of BuMECs. The current dataset on BuMECs proteome will add to the existing information available on mammary proteome and, constitute a reservoir of proteins for further investigation and characterization of the ruminant mammary system in general, particularly the buffalo.

## Results

### Shotgun proteome analysis of sub-cellular protein fractions and conditioned media

The four SCFs which included cytosolic (SCF-I); membranous and membranous organelles (SCF-II); nuclear (SCF-III); and cytoskeletal (SCF-IV)], and conditioned media, from actively proliferating BuMECs were analyzed for identification of proteins using the MS. Data were generated and analyzed separately for each of the five fractions and further combined for the generation of comprehensive proteome profile of actively proliferating BuMECs. A total of 8330 (Table S1), 5970 (Table S2), 5289 (Table S3) and 4818 (Table S4) non-redundant proteins were identified in SCFs (I-IV) respectively with 1% false discovery rate (FDR). The MS analysis of conditioned media identified a total of 792 non-redundant proteins (Table S5), which represent the secretome of actively proliferating BuMECs. Of these proteins 195 were identified with ≥2 peptides. Analysis of 195 proteins using SignalP software suggested that a total of 35 proteins contained a typical signal peptide sequence. The presence of signal peptide sequence demonstrated their secretory nature (Table S6). Altogether, combined analysis of proteins from all the five fractions (SCFs- I to IV, and conditioned media) revealed a total of 12,609 non-redundant proteins in actively proliferating BuMECs (Table S7).

### Gene Ontology (GO), Protein-Protein Interaction (PPI) and Pathway analysis of identified proteins

**Figure 1:**
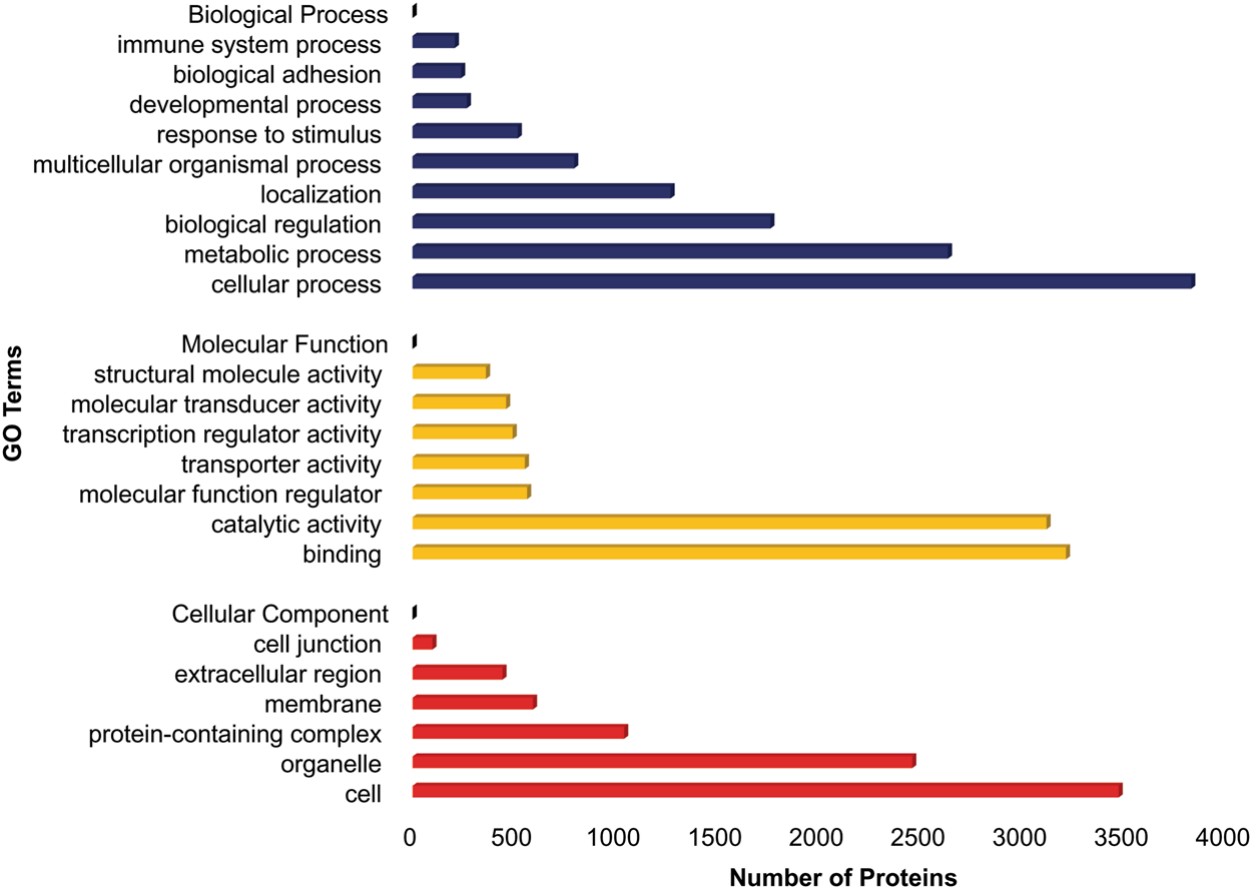
Gene Ontology (GO) classification of the proteins associated with the active proliferation of buffalo mammary epithelial cells (BuMECs). GO analysis divided proteins into three functional groups: Biological Process (BP), Molecular Function (MF) and Cellular Component (CC). Regarding BPs, proteins were mainly involved in cellular and metabolic activities. In terms of the MFs, the major activities associated with proteins were binding and catalytic. The majority of the proteins were present in cell parts, organelles and protein-containing complex.

**Figure 2:**
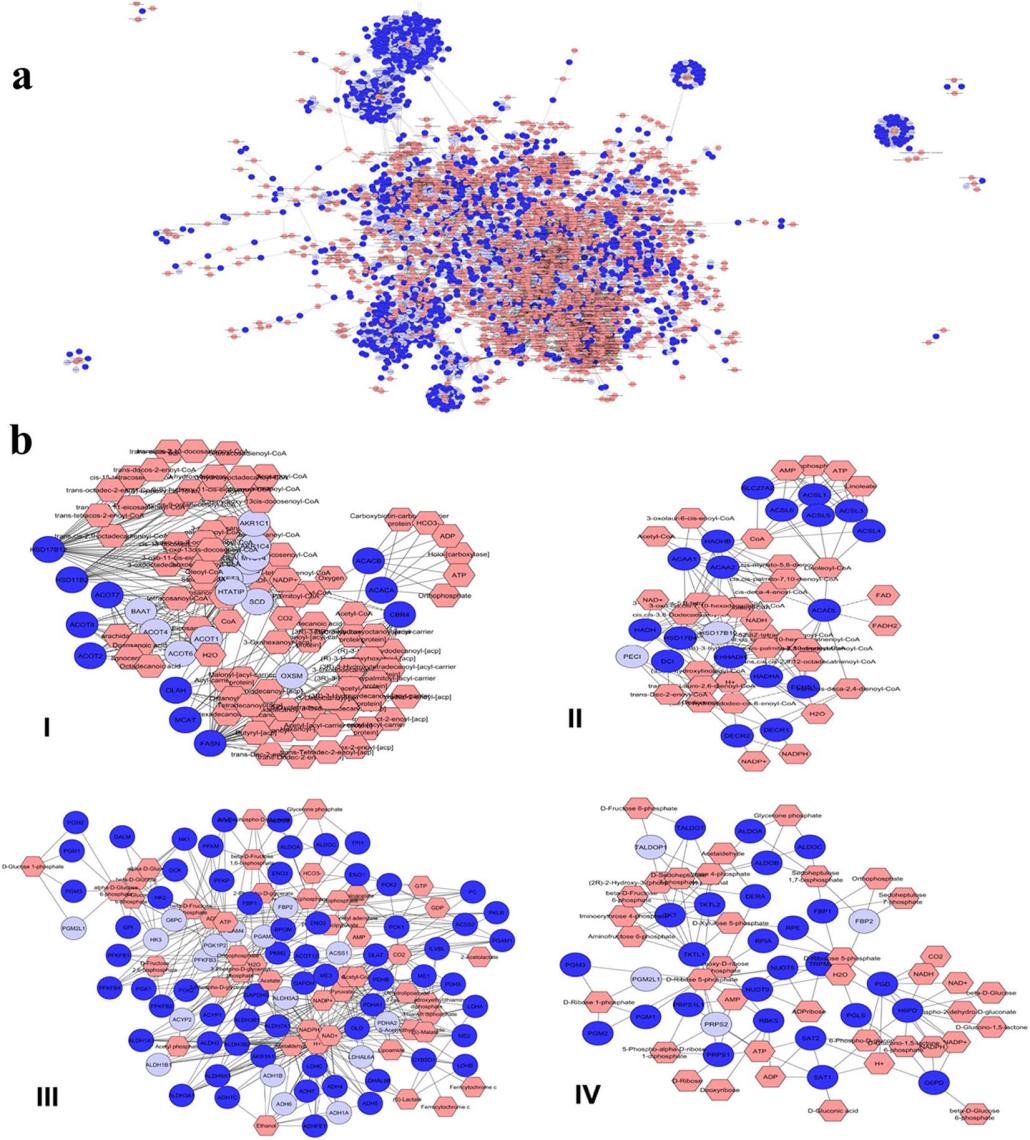
Correlation analysis of proteins and metabolites regulated in actively proliferating buffalo mammary epithelial cells (BuMECs). (**a**) Metscape (Cytoscape Plug-in) analysis generated a complex network comprising input proteins (spherical nodes and dark blue), metabolite (hexagons nodes and neon pink), and reactions (edges). (**b**) **Major metabolic pathways generated**. A total of 92 metabolic pathways were associated with the proteins identified in actively proliferating BuMECs. Selected pathways, including *de novo* fatty acid biosynthesis **(I)**, di-unsaturated fatty acid beta-oxidation **(II)**, glycolysis and gluconeogenesis **(III)**, and pentose phosphate pathway **(IV)** are shown as subnetworks.

Out of total proteome obtained, 10,173 (80.7%) proteins with ≥2 unique peptides were subjected to further Bioinformatics analysis. These proteins represented approximately 63.0% of the protein-coding sequences (CDSs) predicted from the *Bos taurus* genome. GO analysis using Protein Analysis Through Evolutionary Relationships (PANTHER)^12^ categorized the proteins into the biological process (BP), molecular function (MF) and cellular component (CC). The top three GO terms for BP were the cellular process (32.2%), metabolic process (22.1%) and biological regulation (14.8%). In the MF category, the identified proteins were mainly involved in binding (36.5%) and catalytic (35.5%) functions. The GO-based on CC mapped 42.6% and 30.2% of the identified proteins to the cell and organelle, respectively (Fig. 1). The Kyoto Encyclopedia of Genes and Genomes (KEGG) analysis of the proteins was performed to identify the signalling pathways associated with the active proliferation of BuMECs. The results suggested that the proteins were involved in 325 signalling pathways. The pathways such as metabolic, MAPK, RAP1, mTOR, insulin, oxytocin, AMPK, and JAK-STAT with protein counts of 790, 197, 139, 114, 112, 111, 93, and 78 respectively, revealed the protein signatures associated with active proliferation of BuMECs (Table S8). Since a large number of proteins (790) were found to be involved in the metabolic pathways, their roles in metabolism were investigated further using Metscape (Cytoscape Plug-in)^13^. This resulted in the generation of a network based on the known PPIs and Protein-Metabolite Interactions (PMIs) (Fig. 2a). Metscape analysis suggested that the proteins were associated with 92 metabolic pathways (Table S9). Notable pathways included *de novo* synthesis of fatty acids (Fig. 2b-I); β-oxidation of fatty acids (Fig. 2b-II); biosynthesis and metabolism of androgen, estrogen, C-21 steroid hormones, glycosphingolipids (ganglio-, globo- and lacto-series), cholesterol, vitamin B5, glycans (N- and O-) and prostaglandin; glycolysis and gluconeogenesis (Fig. 2b-III); pentose phosphate pathway (Fig. 2b-IV); metabolism of amino acids (biopterin, tryptophan, and tyrosine), vitamins (A, B, D3, E, H, and K) and purine.

**Figure 3:**
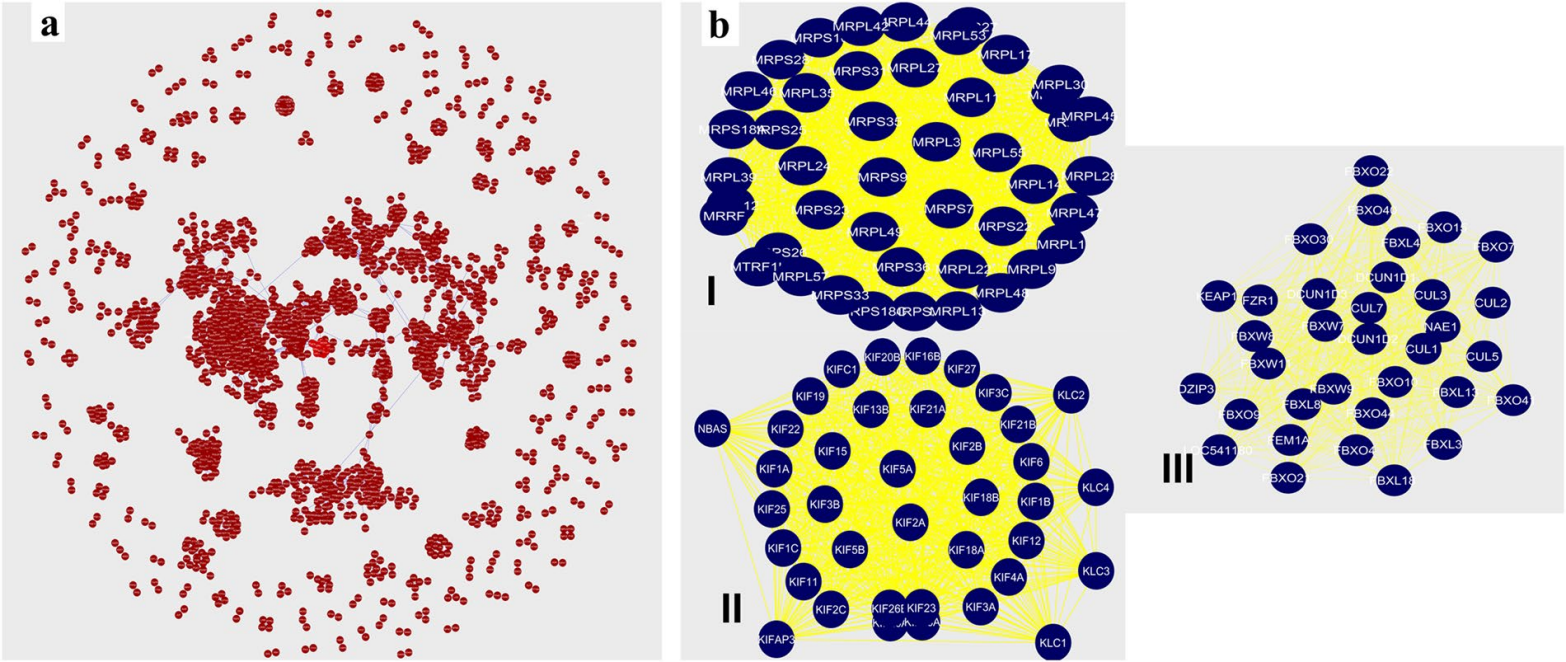
Protein-protein interaction (PPI) network analysis of top 2000 proteins (based on number of peptides identified for each protein) (**a**) The PPI network was constructed using the Cytoscape where each node represents a protein, and edges represent the interaction between two proteins. The PPI network consisted of 1889 nodes and 8813 edges. (**b**) **Molecular Complex Detection (MCODE) analysis**. A total of 138 clusters/modules were extracted from the PPI network using Cytoscape plug-in MCODE. Top three modules with high scores representing Module 1 **(I)** (MCODE score = 44.364, nodes = 45 and edges = 976) suggested the role of proteins in mitochondrial translation, Module 2 **(II)** (MCODE score = 38, nodes = 38 and edges = 703) consisted of proteins having role in intra-Golgi and retrograde Golgi-to-ER traffic, and Module 3 **(III)** (MCODE score = 32.606, nodes = 34 and edges = 538) with proteins mainly involved in class I MHC mediated antigen processing and presentation, and ubiquitin-mediated proteolysis.

The PPIs mediates complex cellular processes^14^. The significance of the PPI network built by the identified proteins was investigated using Search Tool for the Retrieval of Interacting Genes/Proteins (STRING)^15^ version 11.0 (https://string-db.org), and Cytoscape software version 3.6.1 (https://www.cytoscape.org). To achieve this, top 2000 proteins based on peptide number were mapped to the STRING database, which results in the generation of PPI network. From this network PPIs with a high confidence score of ≥0.9 were screened. These interactions were further used to build a PPI network using Cytoscape. The PPI network generated using Cytoscape consisted of 1889 nodes and 8813 interactions (Fig. 3a). The Molecular Complex Detection (MCODE)^16^ analysis of the PPI network, then resulted in the identification of 138 protein modules, also known as clusters (Table S10). Of the 138 modules, the top three modules with the highest scores are represented in Fig. 3b. Module 1 (I) with a score of 44.364, consisted of 45 nodes and 976 edges (Fig. 3b-I). Module 2 (II) with a score of 38, consisted of 38 nodes and 703 edges (Fig. 3b-II). Whereas, Module 3 (III) with a score of 32.606, consisted of 34 nodes and 538 edges (Fig. 3b-III). The pathway enrichment analysis demonstrated that the proteins in module I were enriched with various biological functions such as mitochondrial translation and PPAR-alpha pathway, whereas the proteins in other two modules (-II and -III) were associated with intra-Golgi and retrograde Golgi-to-ER traffic, aurora B signalling, translocation of GLUT4 to the plasma membrane, PLK1 signalling, insulin processing, Class I MHC mediated antigen processing and presentation, and ubiquitin-mediated proteolysis (Table S11).

In the present study, GO analysis of the total identified proteins suggested that the 36.5% protein were associated with the binding functions. The DNA binding proteins regulate the expression of genes during growth and differentiation of the cells^17^. To identify the DNA binding proteins or transcription factors (TFs), we compared the present proteome dataset with the Animal transcription factor database (Animal TFDB) of *Bos taurus* origin (http://bioinfo.life.hust.edu.cn/AnimalTFDB/). The results suggested that a total of 583 proteins were TFs, 261 were transcription-co factors (TCFs) and 102 were chromatin remodelling factors (CRFs) (Table S12). Altogether, 946 proteins were found to have DNA binding functions. The Database for Annotation, Visualization and Integrated Discovery (DAVID)^18^ analysis of DNA binding proteins suggested that most of them (51.6%) were nuclear in origin (Table S13) and were enriched in the transcription process (23.3%) (Table S14).

### GO enrichment, PPI network and Pathway analysis of unique proteins from the SCFs and secretome

**Figure 4:**
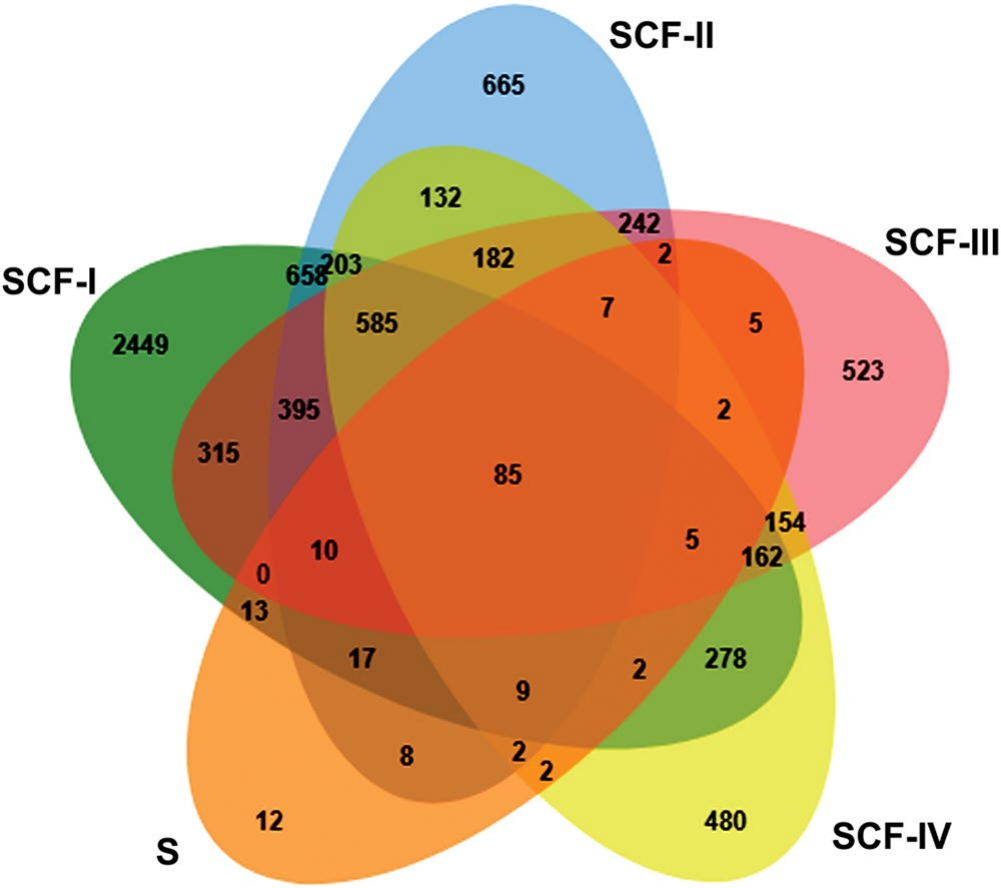
JVENN analysis. JVENN, an interactive Venn diagram viewer software, was used for the comparison of proteome datasets obtained from four sub-cellular protein fractions (SCFs I–IV) and secretome (S). The overlapping area represents proteins shared among the respective fractions. A total of 85 proteins were common among all the fractions.

The proteins (≥2 peptides) unique to sub-cellular compartments (SCF-I to IV) and secretome were identified by comparing proteome datasets obtained for the five fractions. The Venn diagram was constructed using the JVENN tool to display the common and unique proteins among five fractions diagrammatically^19^. The results suggested that 2449, 665, 523, 480 and 12 proteins were unique to the SCF- I, -II, -III, -IV, and secretome respectively (Fig. 4). Whereas, a total of 85 proteins were common among the five fractions (Table S15). Further, DAVID analysis suggested the association of cytosol specific proteins to metabolic, MAPK and PI3-AKT signalling pathways (Table S16); membrane specific proteins to TCA cycle, fatty acid metabolism, and PPAR pathway (Table S17); nuclear specific proteins to MAPK, spliceosome, estrogen signalling and osteoclast differentiation pathway (Table S18); and cytoskeletal specific proteins to ribosome biogenesis, regulation of actin cytoskeleton, axon guidance and SNARE interactions in vesicular transport (Table S19). 35 secretory proteins identified in the conditioned media were involved in the ECM-receptor interactions, focal adhesion, cancer, and TGF-β signalling pathways (Table S20). The proteins common among five fractions were enriched with 23 KEGG pathways. The enriched functions of common proteins mapped mostly to focal adhesion, tight junction, metabolic pathways (Glycolysis/Gluconeogenesis), ECM-Receptor interaction and PI3K-AKT signalling pathway (Table S21).

## Discussion

The proteome signature of an actively proliferating cell conveys in-depth information on the fundamental biological processes associated with its cellular growth and development. MECs are the precursors that are responsible for lactogenesis, and their number is an indicator of milk-producing ability in livestock^20^. Currently, there is very little information available on the proteome profile of MECs of buffalo origin among the livestock species. In the present study, the use of sub-cellular protein fractionation and secretome isolation followed by a shotgun approach identified a total of 12,609 non- redundant proteins which is the most comprehensive proteome profile of any mammalian cell line. The data generated in this study will be useful to understand the mammary gland development in ruminants, particularly in buffalo. Also, the present study will further help to understand lactation biology as the number of MECs in the udder determine the lactation yield of an animal.

Few similar studies have been attempted in MECs from ruminants and other mammals. Jena *et al*.^21^, reported 43 differentially expressed proteins (DEPs) among tissues taken from developing and lactating mammary in buffalo. The Annexin A2 (ANXA2) and peroxiredoxin-6 (PRDX6) were found to be upregulated in Heifer mammary tissue. In contrast, eukaryotic translation elongation factor 1 delta transcript variant 2 (EEF1D), endoplasmic reticulum chaperone BiP (HSPA5), beta-lactoglobulin (LGB), and tissue-specific transplantation antigen P53B (TSTA3) were overexpressed in the lactating buffalo mammary tissue. Janjanam *et al*.^22^ suggested that AKT, PI3K, and p38/MAPK signalling pathways were related to higher milk yield in cattle. Milk fat globule membrane (MFGM) secreted from the apical membrane carries a part of the cytoplasmic fraction of MECs, and they also represent partial information on the proteome of terminally differentiated MECs^23^. Studies on MFGM proteome reported a total of 632, 137, 947 and 1104 proteins in cattle, goat, donkey, and human respectively^24,25^. On the other hand, a proteomics study on mice mammary tissue suggested the biological significance of RNA polymerase B transcription factor 3 (BTF3) in the differentiation of MECs during pregnancy^26^. Cytokeratin 15 (CK15) and dihydropyriminidase-related protein 3 (DRP3) were suggested as markers for the normal breast epithelial cells^27^.

The MECs undergoes proliferation and differentiation based on the physiological status of an animal. Several proteins reported in this study characterized the BuMECs in actively proliferating state. More precisely, the Estrogen receptor (ESR 1 and 2) and various TFs such as proto-oncogene FOS (FOS), cyclic AMP-dependent transcription factor-4 (ATF4), cyclic AMP-responsive element-binding protein 1 (CREB1), CREB3, CREB3L4, specificity protein 1 (SP1), Jun oncogene (JUN), activating transcription factor 2 (ATF2) and ATF6B, which are associated with its signalling were found in the proteome dataset of BuMECs. Estrogen stimulus in a developing mammary gland is important for parenchymal growth^28,29^ and branching morphogenesis^30^. The presence of ESR 1 and 2 in this study suggests that BuMECs have the potential to respond to Estrogen stimulus.

We report for the first time the presence of proteins such as actin-binding LIM (abLIM), secreted phosphoprotein 1 (SPP1), osteopontin (OPN), furry homolog (FRY), and FRY like transcriptional coactivator (FRYL) in BuMECs. abLIMs (isoforms 1–3) function as F-actin binding proteins, which mediates the formation of the actin filament network. They were reported to regulate gene expression, cell-matrix interactions, and cytoskeletal organization. These functions are essential for the cell division, and proliferation^31^. Human isoforms of OPN were reported to impart stemness in MECs^32^. FRY and its paralogue FRYL were suggested to act synergistically in the development and function of kidney^33,34^. The presence of these proteins in our dataset suggests that they may have a potential role during mammary gland morphogenesis in ruminants, which needs further investigation.

We also observed 30 different variants of cytokeratins (CKs) in the proteome of BuMECs. CKs are the major protein components of the intermediate filaments providing mechanical support to the epithelial cells^35^. The expression pattern of CKs was used as a marker to characterize the MECs^36^. Various CKs such as 4, 5, 8, 23, 80 and 78 were reported to be the markers for progenitor cells^37^ and for non-invasive properties^38,39^. Their expression suggests the immortal nature of BuMECs with non-invasive characteristics.

We also report for the first time the presence of ion channel proteins such as Unc-79 homolog, NALC channel complex subunit (UNC79), Unc-80 homolog, NALC channel complex subunit (UNC80), sodium leak channel non-selective protein (NALCN) and aquaporin 6 (AQP6) in the BuMECs proteome dataset. Three proteins, including UNC79, UNC80 and NALCN, are reported to form a cation channel complex in the neurons and myometrium myocytes. The channel is permeable to Na^+^, K^+^ and Ca^+^ ions, by which it regulates the resting membrane potential and excitability in neurons^40,41^. AQP6 is one of the members of AQP family, which includes the transmembrane proteins mainly involved in water transport. However, AQP6 does not have a significant role in water transport. Instead, it exists as an anion channel and mediates the uptake of ions at acidic pH^42^. It is predominantly expressed in the kidney, but its detailed mechanism of action is not well defined^43^. A previous study did not detect AQP6 in bovine mammary gland^44^. Ion channels and aquaporins were reported to be involved in cell proliferation, cell cycle progression, and cell migration, in response to different stimuli^45,46^.

About 40 ATP-binding cassettes (ABC) were found in BuMECs proteome, which included ABCA (11), ABCB (8), ABCC (11), ABCD (3), ABCE (1), ABCF (3) and ABCG (3). A total of 48 ABCs are reported to date, which has been classified into seven families (A to G) based on their amino acid sequences and organization of ATP-binding domains^47^. The number as well as expression of ABCs varies among different tissues in human^48^. ABCs are involved in the transport of cholesterol and other lipids in the mammary gland during lactation^49^.

We identified few germ cell-specific proteins such as testis-specific protein Y-linked 2 (TSPY2), a disintegrin and metalloprotease domain-containing protein (ADAM), ATP-dependent RNA helicase VASA (VASA) and JY-1 in BuMECs proteome dataset. TSPY suppresses p53 mediated apoptotic pathway and enhances the proliferation of spermatogonial cells^50^. ADAMs are transmembrane proteins, which play an important role in the Notch signalling^51^, and various biological activities such as proteolysis, adhesion, and migration^52^. Out of a total of 34 reported proteins belonging to ADAM group^53,54^, 11 members (1, 2, 7, 10, 17, 19, 21, 22, 30, 32, and 33) were identified in BuMECs. VASA is a primordial germ cell (PGC) specific RNA helicase, which play important roles during spermatogenesis and oogenesis^55^. JY-1 is an oocyte-specific marker, which mediates the nuclear maturation and cumulus expansion in oocytes^56^. The presence of these proteins suggests a possible role in the active proliferation of BuMECs, which need further investigation.

In ruminants, the number and secretory activity of MECs determine the milk yield and persistency of lactation^57^. The amount of MECs within the mammary gland depends on their proliferation. The proteome dataset of BuMECs contained a large number of proteins associated with phosphatidylinositol 3-kinase (PI3K)/protein kinase B (AKT) (225 Proteins)^58^, mTOR (114 Proteins)^59^ and nuclear factor-kappa β (NFK-β) (50 Proteins)^60^ signalling pathways, which are reported to have a direct role in the proliferation of MECs. Synthesis and secretion of milk components are among the main functions of MECs. Prolactin^61^, mTOR^62^ and insulin pathways^63^ with a protein count of 57, 114 and 112 respectively, were found in the current dataset. These pathways were previously reported to be involved in the differentiation of MECs. The existence of molecular machinery necessary for differentiation in BuMECs demonstrates the capability of actively proliferating cells to differentiate under the appropriate stimulus. The diverse nature of growth control mechanisms in BuMECs suggests the requirement for complex cellular decisions during various developmental stages of the mammary gland.

Also, the present study reports the presence of endopin, Ca^2+^ dependent activator protein (CAPS2) and Sec 1 family domain containing 1 (SCFD1) in BuMECs. These proteins were previously reported to be associated with secretory functions. Endopin 2 is a serpin and is present in the secretory vesicles^64,65^. CAPS2 facilitates the release of brain-derived neurotrophic factor and neurotrophin from cerebellar granule cells^66^. SCDF1 helps in the release of ECM proteins, which play an essential role in the differentiation of chondrocytes^67^. Further investigation is needed to study the role of these proteins in the BuMECs.

Three variants of aldehyde oxidase (AOX) including 1, 3L1 and 4 were identified in the actively proliferating BuMECs. AOX is a member of the molybdo-flavoenzyme family, which varies in its number and isoforms among mammals such that human express only AOX1, whereas AOX1, AOX3L1 and AOX4 are expressed in cows^68^. The functional significance of these enzymes in MECs is not clear. However, they have been reported to play a role in the clearance of drugs containing aldehydes and N-containing heterocyclic fragments in liver^69^.

Interestingly, we identified 111 proteins in the BuMECs proteome dataset, which were associated with the oxytocin signalling pathway. Oxytocin signalling is usually present in the myoepithelial cells to facilitate the ejection of milk from the alveoli^70^. In contrast, Lolliver *et al*.^71^, reported the presence of oxytocin receptors in ductal MECs in the rabbit mammary gland. The protein kinase C (PKC) identified in our study is a central molecule of the oxytocin signalling. It stimulates the expression of PGF2α through cyclooxygenase 2 (COX2)^72^. Local prostaglandin may affect the blood flow, thereby regulating milk production^73^. Presence of proteins associated with oxytocin pathway in BuMECs, suggests that alveolar epithelial cells have oxytocin responsive mechanisms in milk ejection.

There were 85 proteins common to all fractions of BuMECs. The proteins isolated from various sub-cellular fractions were highly enriched fractions that may have resulted in carryover contamination among the fractions, although some of the common proteins may be ubiquitous. Notable among the proteins found common in five fractions were proliferation associated protein 2G4 (PA2G4) or ErbB3 binding protein-1 (Ebp1), serotransferrin, heat shock protein 10 (HSP10), bromodomain-containing protein 4 (BRD4), and microtubule actin crosslinking factor 1 (MACF1). PA2G4 belongs to a family of DNA/RNA binding proteins which plays an important role in cell growth, apoptosis and differentiation^74–76^. Serotransferrin is a glycoprotein, which is previously reported to have a role in cellular iron transport^77^, growth, differentiation and bacteriostatic effect^78^. HSP10 is a mitochondrial protein which along with HSP60 forms a complex and mainly function in the folding of proteins^79^. HSP10 is reported to act through the RAF pathway and enhances the survival rate of brain cells^80^. BRD4 is a histone binding protein, which plays an essential role in the proliferation of the various cell types, such as squamous epithelial cells^81^, macrophages^82^, and cerebellar granule cell progenitors^83^. In the brain, it acts through sonic hedgehog (SHH) signalling pathway and regulates the levels of effector molecules, including transcription activators- Gli1 and Gli2^83,84^. MACF1 is an actin-binding protein^85^, which is reported to play an essential role in the proliferation of neuronal progenitors through WNT/β-catenin and GSK-3 signalling pathways, during development of the nervous system^86^.

A significant number (372) of uncharacterized proteins were found in the BuMECs proteome dataset. They may represent genes encoding small proteins or novel unannotated genes which require further investigations. These proteins/peptides in BuMECs may have species-specific functions in the mammary gland. The data generated in our study can be used for proteogenomic analysis to identify the non-annotated proteins for their significance in mammary gland biology.

Using an in-depth analysis of the BuMECs proteomics, we identified a large number of DEPs between cell fractions. This information will help to understand the role of identified proteins in mammary gland development and lactation biology. Also, it will serve as a dataset for proteome based genome annotations in buffalo species. The present BuMEC proteome dataset can be used as a reference dataset for parallel comparative studies in other species.

## Materials and Methods

### Cell culture of actively proliferating BuMECs and protein isolation from SCFs

**Figure 5:**
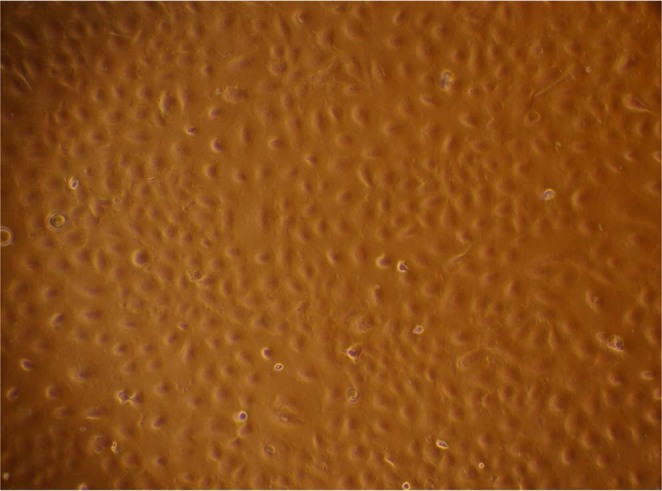
Phase-contrast microscopic image (100×) (Nikon Ti Eclipse, Nikon, Japan) of actively proliferating buffalo mammary epithelial cells (BuMECs). The BuMECs were grown in DMEM supplemented with 10% FBS, 10 ng/ml EGF, 200 mM glutamine, 1 ug/ml hydrocortisone and 5 ug/ml Bovine insulin at 37 °C in a humidified atmosphere of 5% CO_2_. The BuMECs exhibit cobblestone appearance typical of epithelial morphology.

**Figure 6:**
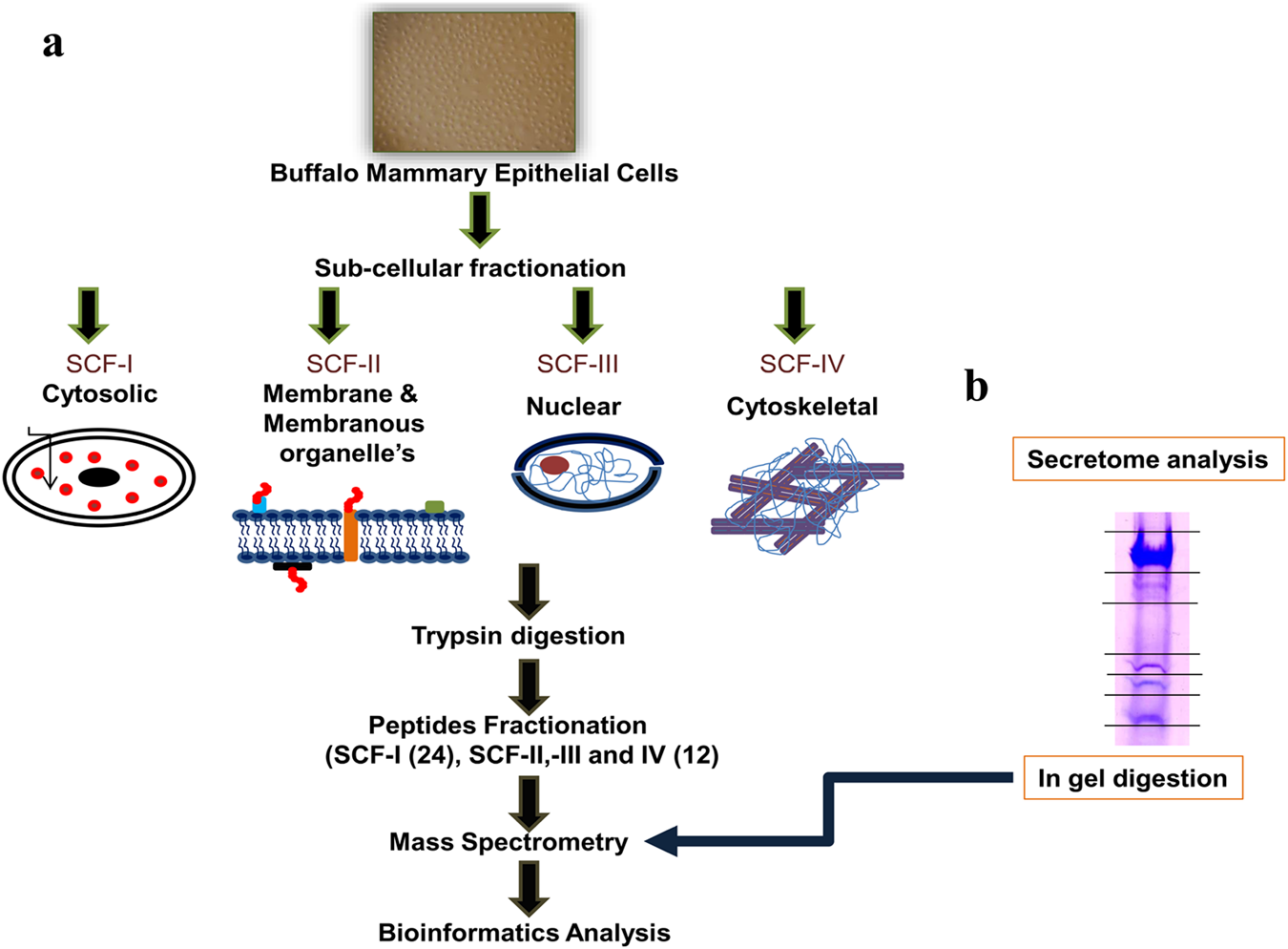
Schematic overview of the analytical workflow. (**a**) Proteomics workflow for sub-cellular protein isolation and MS analysis. The protein from four sub-cellular fractions (SCF-I-IV) was extracted separately from buffalo mammary epithelial cells (BuMECs) using sub-cellular protein extraction kit (SPEK) at 4 °C. The extracted proteins were digested and fractionated using Ultra-High-Performance Liquid Chromatography (U-HPLC) into 24 and 12 individual fractions for SCF-I and SCF (-II-III- and -IV) respectively. Each resultant peptide fraction was analyzed using the nanoLC-MS/MS. The raw data (.mgf files) were analyzed using ProteinScape Software. The biological significance of identified proteins was studied using various Bioinformatics tools. (**b**) **Workflow for secretome analysis**. The protein prepared from the conditioned media was run into the SDS-PAGE (Full-length SDS-Gel image for conditioned media is given as Supplementary Fig. S1), and protein bands were cut into eight gel sections. Each gel section was digested using the In-gel digestion approach and was further analyzed using the nanoLC-MS/MS. Global cellular and secretome analysis identified a total of 12,609 proteins.

The spontaneously immortalized BuMEC line developed in our lab^5^ was used in the present study. Cells at 25^th^ passage were cultivated in Dulbecco’s modified Eagle’s medium (DMEM) (Sigma, US) supplemented with 10% fetal bovine serum (FBS) (Gibco, Thermo Fischer Scientific, US), 10 ng/ml EGF (Sigma, US), 200 mM glutamine (Sigma, US), 1 µg/ml hydrocortisone (Sigma, US) and 5 µg/ml bovine insulin (Sigma, US), at 37 °C in a humidified atmosphere of 5% CO_2_. The cells were grown in T-75 polystyrene flasks (Nunc, Denmark) to 70–75% confluency (Fig. 5). The protein was isolated from the sub-confluent cultures (n = 3) using Proteo Extract Sub-cellular Proteome Extraction Kit (S-PEK, Calbiochem) as per the manufacturer’s protocol. The protein from four SCFs which included SCF-I (Cytosolic proteins), SCF-II (Membrane and Membranous Organelle’s proteins), SCF-III (Nuclear proteins) and SCF-IV (Cytoskeletal proteins) was extracted separately at 4 °C (Fig. 6a). Briefly, the three independent cultures of actively proliferating BuMECs of the same passage were grown (n = 3), and subcellular protein fractions were isolated from these three independent cultures separately. The equal amount of protein from each independent culture was pooled together to generate a single pool for each subcellular protein fraction^87^. The pooled protein fraction for each SCF was then fractionated using ultra-high-performance liquid chromatography (U-HPLC), and processed for deep proteome analysis.

### Clean up of isolated sub-cellular protein fractions and protein estimation

Interfering substances such as detergents, salts, lipids, and nucleic acids were removed from the four protein preparations, which included proteins from SCF-1, -II, -III and -IV, using a 2D-clean up kit (GE Healthcare, US). After cleanup, the proteins were visible as the white pellet, which was then dissolved in 2% diethylamine (DEA). The protein concentration of each sample was estimated using a 2D-Quant kit (GE Healthcare, US) as per the manufacturer’s instructions.

### Cell culture and isolation of proteins from conditioned media for secretome analysis

The BuMECs (25^th^ passage) were maintained as described above and grown up to 70–75% confluency. Subsequently, the media was aspirated, and the cells were rinsed twice with 1X DPBS (Sigma, US) and once with DMEM/F-12, to remove the serum proteins efficiently. The rinsed cells were grown further for 28 h in serum-free DMEM/F-12 media supplemented with 10 ng/ml EGF, 200 mM glutamine, 1 µg/ml hydrocortisone and 5 µg/ml bovine insulin. Conditioned media were collected from each sample (n = 3), and filtered using a syringe filter with a 0.22 μm pore size. The filtered conditioned media was then centrifuged at 1,500 RPM, 4 °C for 10 min, to remove the cell debris. The supernatant was collected and concentrated using a 3 kDa molecular weight cutoff centricon (Millipore, Sigma, US) at 4,000 g, 4 °C. The protease inhibitor (Roche, Switzerland) was added into the protein prepared from the conditioned media and was stored at −80 °C until further processing for MS.

### In-solution tryptic digestion of isolated sub-cellular protein fractions

For In-solution digestion, 365 µg, 200 µg, 165 µg and 120 µg of the protein from four SCFs (SCF-I, SCF-II, SCF-III and SCF-IV) respectively, was lyophilized and reconstituted in 100 mM triethylammonium bicarbonate (TEAB) buffer (Sigma, US). Protein from each SCF was processed separately for trypsin digestion. Briefly, 45 mM dithiothreitol (DTT) (Sigma, US) was added into the protein sample, which was then incubated at 60 °C for 1 h to reduce the disulfide bonds. Subsequently, 10 mM iodoacetamide (IAA) (Sigma, US) was added and the protein sample was incubated in the dark for 30 min to alkylate the cysteine residues. The protein was then digested into peptides by overnight treatment with trypsin (Promega, US) (1:20) at 37 °C. The reaction was subsequently stopped with 10% trifluoroacetic acid (TFA) (Sigma, US), followed which the peptides were vacuum dried and stored at −80 °C until further use.

### Fractionation of digested sub-cellular proteins using U-HPLC

The fractionation of the digested peptides from each SCF was performed using U-HPLC^88^. Briefly, the digested protein sample was loaded onto the C18 column (4.6 × 250 mm, 5 µm, Grace, US) on a Dionex, Quaternary U-HPLC system (Ultimate 3000, Thermo, US) with a UV detection at 214 nm. Two HPLC solvents used were, Solvent A which was 10 mM TEAB and Solvent B which was 10 mM TEAB in 90% acetonitrile (ACN) (Sigma, US). The peptides were fractionated at 25 °C with a flow rate of 1 ml/min and continuous gradient elution (5–100% ACN) from the column over 81 min. The linear gradient was set up as follows: 0 to 2% B for 5 min, 2 to 60% B for 60 min, 60 to 100% B for 10 min, held the same gradient for next 1 min followed by 2% B for 5 min. Digested protein sample from each fraction was run and fractionated separately using the U-HPLC system. A total of 96 time-based fractions were collected and further pooled into 24 and 12 individual fractions for SCF-1 and SCFs (-II, -III and -IV) respectively. The pooling of peptide fractions was done by mixing most hydrophobic ones with most hydrophilic based on concatenation approach. The pooled peptide fractions were then lyophilized, acidified in 0.1% formic acid (FA) (Sigma, US) and desalted using C18 Zip tips (Millipore, US). The desalted peptides were lyophilized and stored at −80 °C before subjecting to nanoLC-MS/MS analysis.

### One Dimensional Gel Electrophoresis (1D-GE) and In-gel tryptic digestion of the protein sample prepared from conditioned media

The concentrated conditioned media was quantified using the 2-D Quant kit as per the manufacturer’s instructions. 25 μg of protein from conditioned media was subjected to 12% SDS-PAGE (10 × 10.5 cm) in a Mini VE complete gel electrophoresis system (GE Healthcare, Sigma, US). The protein bands were visualized by Coomassie brilliant blue (R-350) staining (Fig. S1). The image was obtained using EPSON Scanner (Image ScGE Healthcare, Bio-Science, US). The stained protein bands were then cut into eight equal pieces, and In-gel digestion was performed as reported previously^89^ (Fig. 6b). Briefly, the gel bands were washed with Milli-Q water and destained using 40 mM ammonium bicarbonate (NH_4_HCO_3_) in 50% ACN at a ratio of 1:1 (v/v) followed by rehydration with 100% ACN for 10 min. The protein in destained bands was reduced by 10 mM DTT followed by alkylation using 55 mM IAA. The overnight digestion of reduced and alkylated protein was done with trypsin (1:20) at 37 °C. The reaction was then stopped with 5% TFA, and the digested protein samples were lyophilized. The lyophilized peptides were acidified in 0.1% FA and desalted using C18 Zip-tips following the manufacturer’s instructions. The peptides were stored at −80 °C until nanoLC-MS/MS analysis.

### NanoLC-MS/MS analysis

The fractionated peptides were reconstituted in 0.1% FA, followed which the proteins were identified using captive spray-Maxis-HD qTOF (Bruker Daltonics, Germany) MS with high mass accuracy and sensitivity. The peptides were initially enriched on a nano-trap column (C18, 2 cm, 5 µ, 100 Å, Agilent), followed by elution on to analytical column (15 cm, 3 µ, 100 Å, Agilent). The peptides were sprayed using Nano electrospray emitter tip of 10 µm (Bruker, Germany) using 0.1% FA in water as solvent A, and 0.1% FA in ACN as solvent B. The peptides were loaded onto the trap column using 97% solvent A, followed by resolution on the analytical column using a linear gradient of 5–30% solvent B for 70 min at a constant flow rate of 400 nl/min. The data were acquired in data-dependent acquisition mode subjecting the six most intense ions in each survey scan to MS/MS analysis within a m/z range of 400–2200. The collision-induced dissociation (CID) method was used for precursor fragmentation, and the precursor ions selected for MS/MS fragmentation were excluded after every three spectra. The absolute threshold for precursor ions per 1000 summations was 1200 counts^88,90^.

### Database searching and protein identification

Peak lists were generated by qTOF control (version 24.8) using the Hystar post-processing program to automatically subtract baseline, smoothen peaks and to generate centroid data. The data so generated was searched against the *Bos taurus* database for identification and quantification of proteins using Mascot (2.4.1 Matrix Science, UK) search engine in ProteinScape software 3.2 (Buker Daltonics, Germany)^88,90,91^. Proteins were identified by correlation of mass spectra to entries in the *Bos taurus* database, UniProt (https://www.uniprot.org/)^92^. Mascot MS/MS ion search criteria used were as follows: taxonomy-other Mammalia, specific digestion with trypsin, allowing up to one missed cleavage, MS/MS tolerance of 0.05 Da and peptide tolerance of 50 ppm. The Carbamidomethylation of cysteine was searched as a fixed modification, whereas Carbamyl, Carboxymethyl, Oxidation, and Phospho were set as the variable modifications. The “ion score cutoff” was manually set to 15, thereby eliminating the lowest quality matches. To eliminate the false positives, a 1% FDR was applied at both protein and peptide levels. The raw MS data and the ProteinScape output has been deposited to the ProteomeXchange Consortium (http://proteomecentral.proteomexchange.org) via the PRotemics IDEntifications database (PRIDE)^93^ partner repository with the dataset identifier PXD013707. The secretory proteins in the conditioned media were identified using the web-based Bioinformatics tool, SignalP 5.0 (www.cbs.dtu.dk/services/SignalP/)^94^.

### GO and pathway enrichment analysis of the identified proteins

To evaluate the significance of proteins identified in the BuMECs proteome dataset, GO analysis was done using PANTHER. The proteins were classified into three categories which included BP, MF and CC. To identify the pathways associated with the identified proteins, the KEGG and DAVID, version 6.7 (https://david.ncifcrf.gov) analysis were performed. Fisher exact corrected p-values ≤ 0.05 were considered significant.

### Construction of the PPI network and core protein sub-network analysis

The PPI network analysis was performed using STRING^15^ and Cytoscape. The proteins were mapped to the STRING database, and PPIs with the highest confidence score (≥0.9) were selected. These PPIs were then analyzed using the Cytoscape. The densely connected PPIs or modules were screened out from the main PPI network with the degree cutoff = 2, node score cutoff = 0.2, k-core = 2 and max depth = 100 using MCODE, a Cytoscape plug-in^16^. To better understand the functions of proteins in modules, pathway enrichment analysis was performed using the Reactome Functional Interaction (FI) plug-in 6.1.0.

### Consent for publication

The authors gave their consent for publication of the research results.

## Supplementary information

Supplementary information.

Supplementary dataset .

## Data Availability

RAW MS data have been deposited to the ProteomeXchange Consortium via the PRIDE partner repository with the dataset identifier PXD013707. RAW data were analyzed using the ProteinScape Software, and the output has also been uploaded to the ProteomeXchange Consortium under the same identifier.
