# Supplementary material for: In-depth proteome analysis of more than 12,500 proteins in buffalo mammary epithelial cell line identifies protein signatures for active proliferation and lactation
Source: Sci Rep. 2020 Mar 16;10:4834. doi: 10.1038/s41598-020-61521-1 (PMC7075962; doi:10.1038/s41598-020-61521-1)
Supplement: Supplementary file 1 — Supplementary information. [file 41598_2020_61521_MOESM1_ESM.pdf]

**Supplementary Information:**

**Title:**

# In-depth proteome analysis of more than 12,500 proteins in buffalo mammary epithelial cell line identifies protein signatures for active proliferation and lactation

**Author names:**

Shalini Jaswal[1], Vijay Anand[2], Sudarshan Kumar[1], Shveta Bathla[1], Ajay K. Dang[3], Jai K. Kaushik[1], and Ashok K. Mohanty[1*]

**Affiliations:**

[1] Proteomics and Structural Biology Lab, Animal Biotechnology Center, National Dairy Research Institute, Karnal, 132001, Haryana, India

[2] Department of Veterinary Physiology and Biochemistry, Veterinary College and Research Institute (TANUVAS), Orathanadu, Tamilnadu, 614 625, India

[3] Dairy Cattle Physiology Division, National Dairy Research Institute, Karnal, 132001, Haryana, India

*** Corresponding Author**

**Email Address-** ashokmohanty1@gmail.com

**Postal Address:** Dr. Ashok K. Mohanty (Principal Scientist), Proteomics and Structural Biology Lab, ABTC Center, NDRI, Karnal, 132001, Haryana, India

**Contact information for listed authors:**

libra.88shalinijaswal@gmail.com

vijayanandj6@gmail.com

kumarsudershan@gmail.com

shvetabiotech2010@gmail.com

rajadang@rediffmail.com

jaikr1@gmail.com

**Supplemental Excel files**

**Tables S1 to S21 (Single Excel file as Supplementary Dataset is Uploaded)**

**Table S1:** list of identified proteins in the MS analysis of SCF-I

**Table S2:** list of identified proteins in the MS analysis of SCF-II

**Table S3:** list of identified proteins in the MS analysis of SCF-III

**Table S4:** list of identified proteins in the MS analysis of SCF-IV

**Table S5:** list of identified proteins in the MS analysis of Conditioned media

**Table S6:** List of secretory proteins identified in the conditioned media using SignalP Software

**Table S7:** List of total proteins identified in the comprehensive proteome analysis of four SCFs- I to IV and secretome

**Table S8:** List of the KEGG pathways associated with the total identified proteins

**Table S9.** List of metabolic pathways associated with the total identified proteins

**Table S10:** List of total clusters generated using the Cytoscape plug-in Molecular Complex Detection (MCODE)

**Table S11:** Pathway enrichment analysis of the proteins in the top 3 Modules using Reactome Functional Interaction (FI) plug-in 6.1.0

**Table S12:** List of DNA binding proteins including transcription factors (TFs), transcription co-factors (TCFs) and chromatin remodelling factors (CRFs) among the total proteins identified in BuMECs using the Animal transcription factor database (Animal TFDB) of *Bos taurus* origin

**Table S13:** Cellular Component analysis of the DNA-binding proteins identified in BuMECs

proteome dataset using DAVID

**Table S14:** Biological Process analysis of the DNA-binding proteins identified in BuMECs proteome dataset using DAVID

**Table S15:** List of proteins common among the five fractions (SCFs-1 to IV, and secretome).

**Table S16:** List of pathways significantly associated with the proteins exclusively identified in the SCF-I

**Table S17:** List of pathways significantly associated with the proteins exclusively identified in the SCF-II

**Table S18:** List of pathways significantly associated with the proteins exclusively identified in the SCF-III

**Table S19:** List of pathways significantly associated with the proteins exclusively identified in the SCF-IV

**Table S20:** List of pathways significantly associated with the secretory proteins identified in the conditioned media

**Table S21:** List of pathways significantly associated with the proteins common among the five fractions (SCFs-I to IV and Secretome).

**Supplemental figure**

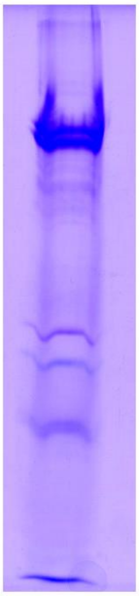

**Figure S1: Coomassie brilliant blue (R-350) stained SDS-PAGE of conditioned media (25 µg protein) representing the Secretome of BuMEC.** The lane with separated protein bands was cut into eight pieces, which were further processed using the In-gel digestion. The digested proteins were identified using nanoLC-MS/MS.
